# Optimising Sprint Performance in Rugby: Insights from a Systematic Review of Training Methods

**DOI:** 10.3390/jfmk10010051

**Published:** 2025-01-29

**Authors:** Manuel Sanz-Matesanz, Francisco Tomás González-Fernández, David Blanco-Luengo, Luis Manuel Martínez-Aranda

**Affiliations:** 1 Faculty of Health Sciences, European University Miguel de Cervantes, 47012 Valladolid, Spain; msanzm@uemc.es; 2 Department of Physical Education and Sports, Faculty of Education and Sport Sciences, Campus of Melilla, University of Granada, 18071 Granada, Spain; ftgonzalez@ugr.es; 3 Faculty of Sports Sciences, Department of Sports and Computer Sciences, Universidad Pablo de Olavide, 41013 Seville, Spain; dblalue@upo.es; 4 Physical Activity Analysis Research Group (SEJ-046), Department of Sport and Computer Science, Universidad Pablo de Olavide, 41013 Seville, Spain; 5 Science-Based Training Research Group (SEJ-680), Physical Performance and Sports Research Center, Universidad Pablo de Olavide, 41013 Seville, Spain

**Keywords:** rugby, sprint training, speed, conditioning, performance

## Abstract

Background: Sprint performance is crucial in rugby, impacting offensive and defensive actions. Despite increasing research on team sports, specific sprint training guidelines for rugby remain limited. This review evaluates the effectiveness of various training methods to improve sprint performance in semi-professional and professional players. Objectives: To identify and assess the most effective training methodologies for improving rugby sprint performance and provide evidence-based recommendations for coaches. Methods: A systematic review adhering to PRISMA guidelines was conducted across PubMed, Web of Science, and SPORTDiscus, including studies published before November 2024. Eligible studies focused on Rugby Union, Rugby League, or Rugby Sevens players undergoing resistance-based or sprint-specific training for at least four weeks. Exclusion criteria included amateur players, athletes under 16, or interventions using advanced technologies. Methodological quality was assessed using the PEDro scale. Results: Twenty-six studies involving 644 rugby players were analysed. Training programmes ranged from 4 to 18 weeks (average: 8 weeks) and were categorised into resistance training, small-sided games, and sprint-specific methods. Resistance training combined with plyometrics and agility drills achieved the most significant sprint improvements. Small-sided games enhanced sprint performance by simulating game-like scenarios, while resisted sprint training showed notable results for short-distance acceleration but limited maximum speed gains. Conclusions: Rugby sprint performance improves through periodised training protocols incorporating resistance exercises, plyometrics, and sport-specific drills. Coaches should use small-sided games and resisted sprint training to target short-distance acceleration and agility. Further research should examine the long-term effects of these methods and their influence on match performance.

## 1. Introduction

The team sports industry, thanks to its high popularity, has allowed for extensive research to be conducted over the years in order to understand the factors and training methodology that most affect sprint performance [1,2]. Sprint performance, or maximum running speed, has been extensively studied due to its significance and has been further segmented into various manifestations. One example is acceleration, defined as the ability to progressively increase speed over a unit of time, with achieving high speeds in the shortest possible time considered a key performance factor [3]. Another example is the ability to repeat sprints, known as Repeated Sprint Ability (RSA), which refers to the capacity to perform high-speed runs continuously at maximum or sub-maximum velocity with minimal recovery time, as typically observed during competition or training [4].

A further key capability studied in relation to sprint performance is agility, which is the ability to execute movements at maximum speed involving changes of direction, often in response to stimuli with high levels of uncertainty [5]. All these manifestations are closely linked to sprint performance, whether in terms of maximum speed achieved during a run, prolonged sprinting, or sprint endurance, which focuses on maintaining a high speed over time without succumbing to fatigue [5].

Given their importance, numerous studies have focused on measuring and developing these aspects within sports performance. It should be highlighted that most studies focused on improving running speed are based on football. Nevertheless, the similarities shared by various team sports, particularly those played outdoors on large fields, allow us to draw conclusions that can be applied to other disciplines, such as rugby or American football [6].

However, there are specific studies that analyse the unique demands of rugby, shedding light on the importance of actions such as sprinting in sports performance. Duthie et al. [7] highlight several key aspects in their systematic review to understand the demands of rugby. Their analysis of movement patterns reveals that of the approximately 5500 m covered by players during a rugby match, 2000 m is spent walking, 1500 m jogging, and 2000 m sprinting, emphasising the critical role of high-speed running in competitive rugby. Additionally, the authors note that the most common sprint distances in rugby range from 10 to 20 m. They further assert that certain professional players can match the performance of specialist sprinters over distances of 15 to 35 m, highlighting the importance of acceleration and speed in high-level rugby.

The most significant finding by the authors, however, is that rugby is one of the sports where a player’s position on the field greatly influences their physical characteristics. Differences in the intensity and type of movement are noted between backs and forwards. Forwards engage in frequent, short-distance high-intensity sprints, while backs tend to spend more time walking and preparing, interspersed with longer sprints when actively involved in play.

It is also worth noting that the differences in high-intensity actions between rugby union and rugby sevens players are not significant. The overall match workload varies due to the different durations of the games, but the total number of high-speed movements is nearly identical. This is because the pace of competition in rugby sevens is higher, with more transitions at very low intensity [8].

Considering the significance of the various sprint manifestations in rugby, it is essential to examine the most common training methodologies used in this discipline. Looking at the most common general characteristics of training methodologies applied to sprinting, the average training duration was 7.4 weeks, consisting of either locomotor resistance training (plyometrics, horizontal jumping, unilateral training) or fixed plane movements (squat jumps and leg extensions) [9].

Along the same line, studies show that physical work in rugby focuses primarily on this type of strength training, which has proven to be an effective methodology for improving running speed [9]. However, there are some doubts among coaches as to whether strength work could lead to hypertrophy, which could be detrimental to players’ speed [10].

The reality of the effects of strength training has been confirmed by studies demonstrating increases in type I and type II fibres’ cross-sectional area in sprinters after long training interventions lasting from 8 weeks to 8 months [11]. It is therefore suggested not to avoid strength training as a whole due to being scared by the increase in fat-free mass (FFM) which can occur after a resistance training protocol [12]. Despite these claims, the benefits of strength training across all sports disciplines far outweigh any adverse effects it may have on athletes. With proper periodisation, training can be directed towards highly beneficial adaptations that enhance an athlete’s speed like changes in muscle architecture like the pennation angle, fascicle length, and region-specific hypertrophy [13,14,15]; nonetheless, resistance training has been shown to increase leg stiffness and ability in small-sided games with consequent positive effects on all sprint performance phases, which are key in rugby [13]. Moreover, beyond specific modifications to sprinting ability, an increase in player mass can offer even greater advantages in rugby. An increase in a player’s mass enhances their momentum during movement (mass × velocity), significantly improving performance during collisions—one of the most common and critical actions in rugby [16].

Additionally, it is important to highlight that rugby is considered one of the sports where players exhibit the greatest differentiation in body composition depending on their position, whether as forwards or backs. This differentiation can sometimes lead to debate regarding the importance of player mass relative to their field position. However, the ability to move at speed and effectively engage in collisions with opponents remains consistently crucial across all positions [7].

Therefore, after acknowledging the importance of training with the specific purpose of increasing sprint performance both in team sports and track and field athletes, the current body of literature lacks guidelines for rugby players. The scientific literature is full of publications analysing rugby players from different points of view, such as performance variables, player physiology, sprint repetition ability, strength and other elements related to general performance. However, specific analysis of one of its most important skills, sprinting, is not so abundant in either the female or male categories [17,18,19,20,21].

Different publications have demonstrated that faster rugby players can break the line, tackles, evade opposing players and score tries more frequently than their slower colleagues; furthermore, being faster, they will engage quicker, carrying the ball, with the defensive line, forcing the opposition into poorer defensive decisions and positioning, thus dominating the contact and creating more tackle breaks [22,23]. Even analysing from the defensive side, faster players make a difference, with a higher tackle score.

Given the evident importance of speed in rugby, supported by empirical data, combined with the variety of training options aimed at improving this ability across different disciplines—with few specifically applied to rugby—this systematic review focuses on compiling the most effective sprint performance enhancement methodologies used exclusively with professional and/or semi-professional rugby players. By reviewing this type of information, coaches will gain objective data on various training methods that ensure a significant impact on players’ performance. This will lead to more efficient training sessions that are specifically geared towards the development of a critical element of the game.

## 2. Materials and Methods

### 2.1. Search Strategy

This systematic review was conducted following the guidelines outlined in the Preferred Reporting Items for Systematic Reviews and Meta-Analyses (PRISMA) recommendations. The scientific databases utilised were PubMed, Web of Knowledge (Web of Science and MEDLINE) and SPORTdiscus. The research considered articles published before November 14th, 2024. The following string was introduced in the above-mentioned databases: (effect* OR change*) AND (“strength training” OR “resistance training” OR “resistance exercise” OR “conditioning program” OR “resisted sled training” OR “training” OR “eccentric training” OR “plyometric training” OR “accentuated”) AND (sprint* OR “sprint performance” OR “speed” OR “acceleration”) AND (“rugby” OR “rugby players”). In addition, reference lists from the relevant articles went through further examination to find other possible sources that fit the inclusion criteria. This protocol/review was not previously registered on any web platform.

### 2.2. Study Criteria

Original studies were included in the systematic review if they included Rugby Union, Rugby League or Rugby sevens players who participated in any kind of resistance-based training intervention lasting at least 4 weeks (considering the minimum time for the changes produced by the training to be due to the proposed intervention), with the main outcome of measuring sprint performance on distances from 5 up to 40 m, which are the most common distances covered by professional rugby players [21,24].

The training protocols included in the review are based on training methodologies commonly used by trainers, excluding protocols that use new technologies, such as electrostimulation or simulated hypoxia devices, as they are considered difficult to apply in practice. Similarly, studies based on systematic reviews were excluded.

Studies were excluded if the sample was composed of randomised team sports players or if the rugby players were clearly stated as amateur or recreational level, and studies with samples under 16 years of mean age were also excluded, since they are considered amateur samples.

### 2.3. Quality Assessment

The methodological quality of the included studies was assessed with the use of the Physiotherapy Evidence Database (PEDro) scale [25]. The PEDro scale consists of 11 items including the specification of eligibility criteria, random allocation strategy, blinded allocation, similarity of groups at baseline, blinding of subjects, therapists (in this case trainers and supervisors) and assessors, intention to treat, between-group analysis, follow-up comparison (85% of individuals reach the post-test analysis), and both point and variability measures. It is important to note that this tool does not measure the validity of the treatment analysed; therefore, a high or low score should not affect the perceived quality of the intervention adopted. Due to the intrinsic characteristics of the studies included, the score ranged between 5 and 7/11.

### 2.4. Data Extraction and Synthesis

After consultation between the authors, manual extraction of the most important data was performed in an excel spreadsheet. The data extracted from the selected studies included the following elements for each article: study; number and level of participants; age (mean yrs ± SD); height (mean cm ± SD); weight (mean kg ± SD); distance (m); intervention duration (weeks); weekly frequency; training type or protocol; tools for measurements; and results. In addition, the tools used to measure the outcome variable, along with the pre- and post-intervention data, were extracted.

To facilitate understanding, an initial table is presented, summarising the previously described variables with clear and concise textual results for easier interpretation. Subsequently, the main findings are organised into categories based on the primary training methods addressed—resistance training, small-sided games, and resisted or assisted sprint training—to further enhance the reader’s comprehension. Additionally, information on *p*-values, means and standard deviations is included where available.

### 2.5. Search Summary

**Figure 1:**
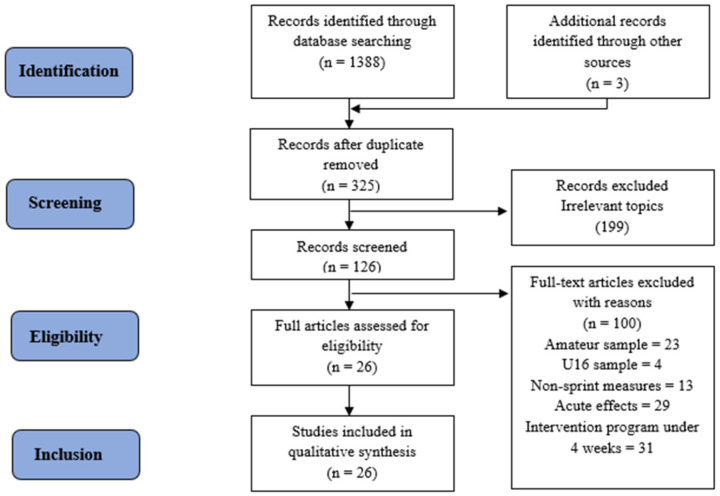
Flow diagram of the article searching process.

The PRISMA methodology was used, consisting of a 27-item scale [26] and a four-phase flowchart (Figure 1). The reviewers MSM and LMMA independently used the search terms to examine the scientific literature through the different selected metasearch engines, such as Pubmed, SPORTdiscus, and MEDLINE. The titles and abstracts of the articles obtained from the initial search were analysed in order to determine the potential articles to be included following the PRISMA flowchart structure (Figure 1). A total of 1388 articles were initially identified through the databases and 3 additional records were found in other sources. After deleting the duplicate articles and carefully reading the abstracts, 199 records were excluded due to irrelevant topics, then 126 were selected for further screening and reading the full text. Then, 100 articles were excluded for not meeting the inclusion/exclusion criteria. Finally, 26 studies were included in qualitative synthesis for this systematic review.

## 3. Results

### 3.1. Characteristics and Main Variables Related to the Studies Included

The pool of studies included in this systematic review is shown in Table 1.

A total of 644 rugby players were investigated. The duration of the interventions ranged from 4 to 18 weeks with an average of ≈8 weeks.

Most interventions consist of two weekly sessions, occasionally including three or four sessions, though these are much less frequent. In nearly all cases, the studies were conducted within a club setting, meaning that external training sessions aimed at technical–tactical development in rugby should be considered.

Only three out of the twenty-six included studies did not find any improvement in sprint performance after a training protocol. Thirteen out of twenty-six found significant and meaningful absolute improvements. Ten studies reported a combination of trivial, non-significant, or no improvements at all, depending on the sprinting distances measured.

### 3.2. Main Results Related to Training Methods

#### 3.2.1. Resistance Training

First, studies focusing on strength, plyometric, and power-based programmes as the core of their intervention are highlighted.

When analysing studies that showed differences but did not reach significance, the wide diversity of methodologies applied becomes apparent. To facilitate analysis, research has been grouped and initially detailed based on mixed training programmes involving strength, plyometric, or power elements, as well as studies focused on training periodisation.

An example is the work by Shattock and Tee [45], which compared two methodologies for strength and speed training in 20 semi-professional rugby players (mean age 22.5 ± 3 years). The initial training block was regulated using technology that provided objective feedback to the player, followed by a block guided by the subject’s subjective perception. The changes were trivial in both cases, with better outcomes for the group following objective training regulation (0.4% improvements in sprints compared to 0.1% for the subjective group, with no significant results). In a similar vein, Harries et al. [41] compared linear and undulating strength training periodisation in 26 junior players (aged 15–18 years) from the British national talent programme. The 12-week block yielded trivial changes that did not reach significance, but a clear advantage was observed with the undulating method (0.045 s reduction in the undulating group, 0.029 s in the linear group, and 0.024 s in the control group over 10 m; 0.051 s in the undulating group, 0.016 s in the linear group, and 0.005 s in the control group over 20 m). Similarly, McMaster et al. [37] studied the differences between two complex training methodologies: strength training plus light ballistic load (15/30% 1RM) and strength training plus heavy ballistic load (60/75% 1RM). After a 10-week intervention involving 14 semi-professional rugby players (mean age 21 ± 1.6 years), sprint time improvements were found, though they were not significant for either method [10 m (−1.2 to −1.6%), 20 m (−1.0 to −1.2%), and 30 m (−0.6 to −0.9%)].

Finally, two studies focused on periodised training interventions, examining the effect of a tapering period following a phase of high-intensity training. Marrier et al. [39] conducted a three-week tapering period after four weeks of progressive training involving rugby-specific actions, strength training, and high-intensity exercises with 10 international Rugby sevens players (mean age 26 ± 5 years). Post-taper, the players reduced their sprint times (−3.1% ± 0.9%), improving performance, though results did not reach significance. Coutts et al. [29] applied a protocol involving deliberate overtraining for six weeks followed by a one-week tapering period to seven semi-professional rugby players (mean age 25.7 ± 2.6 years). The protocol incorporated field-specific work, strength, cardiovascular training, and agility exercises. After the seven weeks, trivial improvements were found in the 10 m (pre-training 1.89 ± 0.09 s, post-training 1.92 ± 0.11 s, and post-tapering 1.88 ± 0.10 s) and 40 m (pre-training 5.42 ± 0.18 s, post-training 5.46 ± 0.20 s, and post-tapering 5.44 ± 0.19 s) tests.

Secondly, various studies employing specific strength training methodologies without periodisation but failing to achieve significant differences are noteworthy.

Harris et al. [30] compared two strength training methodologies in 18 professional rugby players (mean age 21.8 ± 4 years): one at 80% 1RM and another allowing for maximum power output (around 40% 1RM). No significant benefits were found in either case. Similarly, Loturco et al. [52] used squat jump training with load in elite rugby players (mean age 25.4 ± 2.7) over four weeks, comparing 40% 1RM versus 80% 1RM protocols, and reported no significant differences (*p* > 0.05), except for players’ perceived exertion (*p* = 0.013). Appleby et al. [47] studied 33 experienced players, comparing bilateral and unilateral training, finding trivial improvements in 20 m sprint speed with no differences between groups (BIL = −0.38 ± 0.49 s; UNI = −0.31 ± 0.31 s). Similarly, Weakley et al. [44] compared two groups of semi-professional players, one receiving feedback during training (*n* = 16) and the other without feedback (*n* = 12), finding no significant differences between the groups. Additionally, Pienaar and Coetzee [34] investigated 35 South African U19 rugby players, comparing an experimental group (*n* = 19) that incorporated plyometric training into strength sessions against a control group (*n* = 16). While the experimental group outperformed the control group, data remained trivial in 5 m (sprint 5 m (s): control pre 1.14 ± 0.17, post 1.14 ± 0.18; experimental pre 1.22 ± 0.16, post 1.14 ± 0.1), 10 m (sprint 10 m (s): control pre 1.90 ± 0.19, post 1.90 ± 0.16; experimental pre 1.98 ± 0.18, post 1.92 ± 0.12), and 20 m (sprint 20 m (s): control pre 3.22 ± 0.24, post 3.26 ± 0.19; experimental pre 3.34 ± 0.25, post 3.25 ± 0.16).

Other examples include Orange et al. [43], who compared two strength training individualisation methods in professional U18 players (mean age 17 ± 1 year): velocity-based training (*n* = 12) versus percentage-based 1RM training (*n* = 15). They found trivial, inconclusive differences in sprint data for 5, 10, 20, and 30 m after seven weeks of intervention. Simpson et al. [49] applied a strength training method based on individual force–velocity profiles and imbalances in squat jumps to 29 professional players (mean age 24 ± 3 years), resulting in improved vertical jump tests (40 to 45 cm), but without significant horizontal transfer or differences in 10 or 20 m sprint tests. Similarly, Gabbett [27] implemented an exercise programme aimed at injury control and performance enhancement in rugby, comparing senior (*n* = 41) and junior (*n* = 36) samples. Although significant improvements in parameters like Vo2Max were achieved, the limited specific volume dedicated to sprint training meant no improvements were seen in 10, 20, or 40 m sprints.

Lastly, studies achieving significant results with strength-based training programmes deserve mention.

Among these are the studies by Comfort et al. [33], which combined 4 weeks of strength training with a further 4 weeks of agility, plyometrics, and power-specific work applied to 19 professional rugby players. Significant improvements were observed in 5 m sprints (pre: 1.05 ± 0.06 s; post: 0.97 ± 0.05 s), 10 m sprints (pre: 1.78 ± 0.07 s; post: 1.65 ± 0.08 s), and 20 m sprints (pre: 3.03 ± 0.09 s; post: 2.85 ± 0.11 s). Similarly, McLaren et al. [40] incorporated strength, power, agility, and speed training sessions, combined with a tapering period, in an intervention involving 23 professional players (mean age 24 ± 3 years). Significant improvements were recorded over 10 m (pre 1.79 ± 0.10 s, post 1.68 ± 0.07 s), 20 m (pre 3.13 ± 0.17 s, post 3.02 ± 0.14 s), and 30 m (pre 4.34 ± 0.11 s, post 4.22 ± 0.10 s) sprints. In a similar vein, Randell et al. [32] conducted a squat jump-based training programme with 13 professional rugby players, providing feedback to one group (*n* = 7) and no feedback to the other (*n* = 6), finding improvements between 0.9% and 1.4% over 10, 20, and 30 m for the feedback group, with significant results (*p* = 0.0008) for the 30 m distance.

Regarding specific training methodologies, several notable studies include that of Douglas et al. [42], who examined the effects of accentuated eccentric training applied to the squat exercise in 14 experienced players (mean age 19.4 ± 0.8 years) to enhance sprint speed. Results indicated greater benefits when the eccentric phase was three times longer than the concentric phase (pre 5.41 ± 0.14 s, post 5.36 ± 0.13 s) compared to an equal pace for both phases (pre 5.41 ± 0.14 s, post 5.44 ± 0.12 s) in 40 m sprints. On the other hand, Speirs et al. [38] analysed differences between unilateral and bilateral strength training and their impact on sprint performance in a sample of 18 developing players (mean age 18.1 ± 0.5 years). The study showed improvements in both groups only over the 40 m distance (unilateral pre 5.35 ± 0.15 s, post 5.26 ± 0.16 s; bilateral pre 5.40 ± 0.26 s, post 5.34 ± 0.23 s). Similarly, Scott et al. [51] compared the effects of two complex training programmes (traditional and variable-resisted) with a control group among university-level rugby players. The results indicated significant changes over 5 m in both training groups (*p* < 0.05) and significant improvements over 10 (*p* = 0.029) and 20 (*p* = 0.006) metres in the traditional complex training group. Lastly, Zabaloy et al. [48] focused on strength training using the force–velocity profile and its imbalance calculation relative to theoretical optimisation. In a sample of 34 experienced players, they found significant sprint improvements after common strength protocols, ranging from 0.01 to 0.03 s over 5, 10, 20, and 30 m. More pronounced improvements of up to 0.06 s were achieved in groups that individualised training based on their profile.

#### 3.2.2. Small-Sided Games

Beyond conventional strength training methodologies, two training approaches yield highly positive results in rugby sprint performance: modified specific training such as small-sided games, and speed training using assisted and resisted running.

Gabbett [28] conducted a study comparing two training protocols applied to 69 elite players: a traditional programme of sprints, agility, and power (*n* = 37, mean age 22.3 ± 0.8) and one based on simulated game situations aimed at improving player agility and speed (*n* = 32, mean age 22.1 ± 0.9). The results indicated that the group trained in simulated game situations achieved sprint time improvements over 10 (pre 1.91 ± 0.02 s, post 1.81 ± 0.02 s), 20 (pre 3.17 ± 0.02 s, post 3.07 ± 0.02 s), and 40 (pre 5.64 ± 0.03 s, post 5.47 ± 0.03 s) metres, while the traditional training group only improved over 10 metres (pre 1.85 ± 0.01 s, post 1.80 ± 0.01 s). In a similar study, Seitz et al. [36] supplemented strength and preventive training with two sessions of small-sided games for 10 elite players (mean age 20.9 ± 1.4). Notable sprint time improvements were observed over 10 (pre 1.95 ± 0.07 s, post 1.89 ± 0.06 s), 20 (pre 3.28 ± 0.10 s, post 3.24 ± 0.08 s), and 40 (pre 5.34 ± 0.16 s, post 5.28 ± 0.13 s) metres, while controlling for other speed-focused training.

#### 3.2.3. Resisted or Assisted Sprint

The use of speed training methodologies involving resisted or assisted sprinting is very common in rugby. Their application is supported by studies such as that by West et al. [35], which compared a sample of 20 elite players divided into two groups: one performing traditional sprint training and the other using a sled with a load equivalent to 12.6% of the player’s body mass. The results showed improvements in both groups, with greater significance in the group using sled-resisted sprinting (10 m sled pre 1.74 ± 0.10 s, post 1.70 ± 0.10 s vs. traditional pre 1.74 ± 0.07 s, post 1.72 ± 0.06 s; 30 m sled pre 4.26 ± 0.28 s, post 4.15 ± 0.18 s vs. traditional pre 4.19 ± 0.19 s, post 4.15 ± 0.18 s).

Similarly, Lahti et al. [46] compared the improvements from sled-resisted sprint training at 75% of the player’s maximum speed (Vmax) in a sample of 6 players (mean age 19 ± 0.3 years) with assisted sprinting at 105% of their Vmax in a sample of 10 players (mean age 20 ± 1 year). It was found that only the resisted training group achieved improvements in 20 m sprints (pre 3.44 ± 0.11 s, post 3.32 ± 0.08 s).

Continuing with the use of sleds, the last two articles in this review provide contrasting data regarding the differentiating capacity of sled training compared to common training. Harrison and Bourke [31] investigated the effectiveness of sled training with a load equal to 13% of the participant’s body mass, comparing it to a control group (mean age 20.5 ± 2.8 years, *n* intervention = 8, *n* control = 7). Significant improvements were found in the experimental group in skills such as jumping, along with better 5 m sprint times within a 30 m sprint (*p* = 0.020). However, no significant differences were found over longer distances, highlighting the effectiveness of this training for static starts, which are common in rugby.

Conversely, Sinclair et al. [50] compared a sample of 28 professional players (mean age 18.8 ± 0.6 years) divided into two groups: one trained with resisted sprints (*n* = 13) and the other with normal sprint training (*n* = 13). The results showed significant performance improvements without differentiation between the groups (5 m pre 1.03 ± 0.07 s, post 0.97 ± 0.08 s; 10 m pre 1.77 ± 0.06 s, post 1.70 ± 0.06 s; 20 m pre 3.01 ± 0.10 s, post 2.94 ± 0.11 s), with differences found only in agility and jump tests, as also noted in the study by Harrison and Bourke [31].

## 4. Discussion

The main objective of this systematic review was to examine the training methods commonly used in rugby clubs and investigated in research studies. Furthermore, the most effective methods were evaluated, in order to be able to provide viable and practical applications for strength and conditioning coaches working in the semi- and professional rugby industries. This review specifically focused on studies analysing non-amateur players, being either elite academy level, semi-pro, professionals, or even elite athletes.

Among the articles that provide methodologies with positive changes in players’ sprint performance, it is important to highlight that, although many of the authors manage to control all the training variables, in some cases, the study itself mentions that some of the improvements in sprint times may be due to multiple factors, not just the intervention protocol, and are not entirely clear. These statements are because the players are in competition season, and that technical–tactical training is also conducted alongside physical training, which may influence the improvement of the results. Similarly, complementary physical training sessions are carried out, which can also affect the performance of the sample due to interference with training adaptations [33,42,53]. This statement is crucial when selecting protocols carefully and studying their effectiveness in depth.

### 4.1. Resistance Training

Of the studies included in this review, 17 of them analysed resistance training-based protocols and 8 of them found clear improvements. As the results of the studies suggest, the best improvements came from well-designed protocols considering the already-in-place rugby schedule.

The improvements achieved through strength training associated with sprint velocity are based on different methodologies. The combination of strength work with loads and plyometric and agility training seems to be the most suitable for enhancing sprint performance, benefiting from sport-specific strength work related to actions typical of the sport [33,40]. Strength training yields positive results when programmed alongside agility work, but also when performed in isolation, provided that the loads are properly organised and individualised work is pursued, as demonstrated in the study by Zabaloy et al. [48]. Similarly, the application of modifications to strength training, such as the selection of predominantly unilateral exercises [38,47], the use of feedback during strength training [44], the incorporation of complex training methods [51], or the use of accentuated eccentric phases [42], has proven to be useful for improving sprint speed. Based on this, the use of these strength training options, when programmed in conjunction with agility training, can be highly beneficial for the development of sprinting capacity in rugby players. These programmes have demonstrated their usefulness when applied to other sports, which further justifies their relevance to rugby [54,55].

It is important to note that individualised work based on the force–velocity (F-V) profile has become a significant area of interest in recent years, providing benefits in team sports related to vertical actions [56]. However, its connection with the transfer of a methodology for calculating force along the vertical axis to a horizontal action is highly controversial. Several studies assert that the inclusion of sprints and horizontal strength exercises is necessary to achieve improvements in players’ speed. With a programme focused on improving the squat profile, performance gains are typically linked to jumping, but not necessarily to running [49,56,57]. For this reason, several studies included in this review failed to produce significant results in improving rugby players’ sprinting performance, all attributing this to the lack of specific sprint training in the strength programme. Practising the specific action to be improved is crucial in achieving effects, rather than relying solely on parallel training [27,43,49].

Similarly, not only should the content of strength sessions be highlighted, but also their periodisation over time. In this case, the application of undulating methodologies, where intensity is varied in each session without a constant progressive increase, represents an improvement over traditional periodisation, although the results do not show strong or significant differences [37,41]. These results, with no significant differences, align with findings from studies in football or handball, where despite the evident improvement, there is no real difference between undulating periodisation methods and traditional ones [58,59].

In addition to the orientation of strength training periodisation, the importance of controlling intensity must be emphasised. Several studies suggest that applying high-intensity sessions without adequate control, even with a tapering phase, can result in negative outcomes. Therefore, meticulous control of intensity and a subsequent de-loading phase are recommended to ensure proper supercompensation in players, similar to what is applied in other disciplines [29,39,60].

### 4.2. Small-Sided Games

The use of simulated game situations in the development of sprint speed in rugby is highly beneficial, with improvements being linked to an increase in player motivation through the completion of tasks during training, far surpassing the motivation achieved through traditional methods [28,36]. The advantages also lie in a greater transfer of training situations to real competition, as simulated games involve actions that cannot be carried out with traditional training methods [28]. These findings align with those reached in various other sports disciplines, such as football [61] or basketball [62], where small-sided games have been found to be an alternative to traditional methods for developing key game skills, while also providing a motivational element for players. However, all authors stress that the programming of such training should be studied in greater depth.

### 4.3. Resisted or Assisted Sprint

As stated in previous sections, specific sprint training is essential for its improvement, and the use of training methodologies focused on this action that aim to enhance the movement through drag or assistance is commonly employed by coaches.

The performance improvements in sprinting achieved through the use of sleds have been demonstrated in studies applied to rugby [35,46], but they are also common in other disciplines such as football [63] or swimming [64]. However, in all these studies, it is noted that the potential benefits should be approached with caution, as comparisons with sprint training without assistance or resistance show minimal or non-existent differences. Benefits are found in actions related to sprinting, such as change of direction and agility, but not in maximum speed achieved [31,50].

Therefore, it should be noted that if the focus of training is exclusively on improving linear sprinting, the use of sleds or assisted running elements may not be necessary, as pointed out in previous studies. However, given the benefits that resisted sprints bring to other key variables for rugby performance, such as change of direction, their use is justified [50]. Additionally, a possible beneficial effect linked to the use of sleds in sprinting actions is post-activation potentiation, related to the use of loads during running, which is not present in unloaded sprint training and may influence the acute training effects on player performance [35]. Furthermore, the relevance of using heavy sleds (>40% body mass) as a method of horizontal strength training is highlighted, which could be of interest for rugby players’ linear movements [65].

This systematic review provides valuable insights into the effectiveness of different training methods to improve sprint performance in rugby players; however, several limitations should be acknowledged. Firstly, it should be emphasised that the studies analysed in the present review specifically focused on professional or semi-professional rugby players. This condition makes it impossible to segment the sample into control and intervention groups, as such an approach is considered unfeasible and unethical within high-level competitive structures. It is not viable to generate positive effects for only part of the team without extending them to the entire group.

As a result, and due to the impossibility of including control groups, some of the positive effects observed following the implementation of training programmes may not be solely attributable to the intervention itself but rather to other external variables. Similarly, the number of included studies and the overall sample size were limited, particularly in certain training methods, which may restrict the generalisability of the findings to wider rugby populations. Additionally, the methodological heterogeneity among the included studies—such as variations in training protocols, the use of mixed training methodologies, differences in intervention durations, and participant characteristics—complicates the identification of the specific method responsible for the observed positive effects. This variability also makes direct comparisons challenging and may affect the robustness of the conclusions. Furthermore, the studies primarily focused on the short- to medium-term effects of the training interventions, leaving the long-term impact of these methods on sprint performance underexplored. Most studies were conducted within controlled club or training settings, which may limit the applicability of the findings to real-world competition environments, where external factors could influence performance outcomes. Finally, interventions involving advanced technologies, such as electrostimulation or simulated hypoxia, were excluded from this review. While this approach ensures practical applicability, it limits the exploration of potentially impactful but less accessible training methods. Future research should aim to address these limitations by including larger and more diverse samples, standardising training protocols, and evaluating the long-term effects of interventions under competitive conditions to provide a more comprehensive understanding of the optimal strategies for enhancing sprint performance in rugby players.

## 5. Conclusions

Sprint performance in rugby players has been widely examined and investigated using different training modalities. As shown in the studies included, if the research wants to investigate professional and elite athletes, it is necessary to consider that different, intensive training sessions or even weekly competitions may take place during the intervention period. In fact, overcoming this big limitation, the studies that resulted in sprint performance improvement are those that designed a protocol around the pre-existing schedule, periodising it accordingly.

From this review, it is possible to conclude that an effective pre-season conditioning plan should include a resistance training protocol, in a periodised fashion, decreasing volume and increasing intensity over the pre-competitive weeks, to taper the accumulated fatigue. The protocol should follow a traditional structure combined with power, agility and plyometric work, thus ensuring optimal adaptations for the player. It is clear, though, that an excessive volume of concurrent conditioning sessions or weekly competition can interfere with the positive adaptations of RT.

Rather than separating traditional skills and conditioning sessions, a combination of both into weekly sessions of small-sided games can be greatly beneficial to sprint performance, especially if administered with the proper intensity and volume. Finally, adding resisted sprint sessions, with a load of around 13% of body mass, could be the key to improving specific sprint performance in professional rugby players.

## Figures and Tables

**Table 1:** Main variables related to the studies included in the systematic review.

Study	Number and Level of Participants	Age (Mean yrs ± SD)	Height (Mean cm ± SD)	Weight (Mean kg ± SD)	Distance (m)	Duration (Weeks)	Weekly Frequency	Training Type	Tools for Measurements	Results
Gabbett [27]	36 sub-elite	16.9	176.3	74.3	10–20–40	14	2	GPP and specific preparation	Dual-beam electronic timing gates	No significant differences between groups nor pre- to post-training for sprint performance
	41 sub-elite	25.5	177.9	89.8	10–20–40	14	2	GPP and specific preparation		
Gabbett [28]	37 sub-elite	22.3 ± 0.8	-	-	10–20–40	9	2	Traditional conditioning	Dual-beam electronic timing gates	Significant improvement in 10, 20, and 40 m sprint times (skill), 10 m sprint (traditional). Significant difference in 20 and 40 m (skill > TRAD)
	32 sub-elite	22.1 ± 0.9	-	-	10–20–40	9	2	Skill-based conditioning		
Coutts et al. [29]	7 semi-pro	25.7 ± 2.6	176	86.1 ± 10	10–40	6	3	Rugby conditioning + RT, aerobics, skills, sprint sessions	Electronic timing gates	Only performance over 10 m reached a minimum clinically important difference from pre-training
Harris et al. [30]	7 elite	21.8 ± 4	180.7 ± 4.6	96.2 ± 9.9	10–30	7	2	Percentage-based Explosive RT	Kinematic measurement system	Improvements over 10 and 30 m sprint times (percentage-based optimal). Neither of the changes was statistically significant
	8 elite	-			10–30	7	2	Optimal load explosive RT		
Harrison and Bourke [31]	7 pro and semi-pro	20.5 ± 2.8	-	87 ± 10.5	10–20	6	2	Sled-resisted sprint + RT + speed	Laser	Significant improvements in 5 m (resisted sprint). Similar mean improvements in 10 and 30, without statistical significance
	8 pro and semi-pro	-	-	-	10–20	6	2	RT + speed		
Randell et al. [32]	7 pro	25.7 ± 3.6	188.5 ± 8.2	104.3 ± 10	10–20–30	6	3	RT with feedback	Electronic timing gates	Significant improvements in 30 m. Training with feedback produces a positive effect on the players in their effort during the practice, being superior to training without feedback
	6 pro	24.2 ± 2.5	184.7 ± 7.2	102.9 ± 14.3	10–20–30	6	3	RT without feedback		
Comfort et al. [33]	19 elite	-	184 ± 6	96.2 ± 11.1	5–10–20	8	2	Undulated strength and power training + plyometrics	Infrared timing gates	Significant improvements in 5, 10, and 20 m sprint times
Pienaar and Coetzee [34]	16 university	18.94 ± 0.38	-	-	20	4	3	Rugby conditioning + RT + plyometrics	Intermediate beam photocell timing system	Experimental sprint times over 5, 10, and 20 m were definitively decreased, while significant statistical difference was found only for 20 m distance
	19 university	18.94 ± 0.42	-	-	20	4	3	Rugby conditioning + RT		
West et al. [35]	10 pro	26.8 ± 3	186 ± 8	90.2 ± 10.3	10–30	6	2	Sled-resisted sprint	Electronic timing gates	Significantly decreased sprint times over 10 and 30 m, with greater improvements for the sled-resisted sprint group.
	10 pro	25.1 ± 3.2	185 ± 7	90.9 ± 10.6	10–30	6	2	Traditional sprint		
Seitz et al. [36]	10 elite	20.9 ± 1.4	184.7 ± 7.4	94.4 ± 8.6	10–20–40	8	2	Small-sided games + skills + RT	Electronic timing gates	Significant improvements were made in 10, 20 and 40 m sprint times
McMaster [37]	11 county-level	20.9 ± 1.6	185 ± 5	95.2 ± 7.4	10–20–30	10	4	CPX: Strength + light ballistic strength + heavy ballistics	Dual-beam infrared timing lights	Small to moderate decreases over 10, 20, and 30 m sprint times
Speirs et al. [38]	9 academy	18.1 ± 0.5	183 ± 3.4	96.7 ± 9.3	10–40	5	2	Unilateral RT + skills + conditioning	Electronic timing gates	Significant improvements only for 40 m sprint, similar for both groups. No significant changes for 10 m distance
	9 academy	18.1 ± 0.5	185 ± 8.9	98.1 ± 13.4	10–40	5	2	Bilateral RT + skills + conditioning		
Marrier et al. [39]	10 elite	26	179 ± 9	90 ± 11	30	7	4	Rugby conditioning + RT + high-intensity training	Timing system	Unclear change in 30 m sprint time after the 4-week block, while during the taper, the decrease in time was certain large
McLaren et al. [40]	23 pro	24 ± 3	181 ± 17	100 ± 13	10–20–30	8	4	GPP and specific preparation: HIIT + RHIE + RT + skills + speed	Photo electric timing gates	Improvements in 10, 20, and 30 m sprint, with a likely large, possibly large, and likely moderate inference for the respective distances
Harries et al. [41]	8 academy	16.8 ± 1	180.4 ± 3.3	88.6 ± 18.2	10–20	12	2	Rugby + linear periodisation RT	Electronic timing system	Moderate and small decreases in 10 and 20 m sprint times (linear); small but significant decreases in both distances (undulated). Control group only showed a small decrease in 10 m sprint
	8 academy	17 ± 1.1	181.3 ± 7	82.4 ± 12.6	10–20	12	2	Rugby + undulating RT		
	10 academy	15.5 ± 1	174.3 ± 5.4	69.9 ± 8	10–20	12	-	Rugby (control)		
Douglas et al. [42]	7 academy	19.4 ± 0.8	182 ± 5	97 ± 11.6	10–20–40	8	3	Traditional RT	Radar device	Slow eccentric AEL training is superior to slow eccentric traditional training
	7 academy	-	-	-	10–20–40	8	3	Accentuated eccentric loading RT		
Orange et al. [43]	15 academy	17 ± 1	181 ± 6.3	84.9 ± 11.9	5–10–20–30	7	2	Percentage-based RT	Photocell timing system	Likely and very likely decline in sprint performance
	12 academy	17 ± 1	178 ± 5.3	81.8 ± 11.9	5–10–20–30	7	2	Velocity-based RT		
Weakley et al. [44]	16 semi-pro	21 ± 1	185.9 ± 6.2	98.4 ± 13.1	10–20	4	3	Sprint + RT with feedback	Electronic timing gates	Between-group differences were unclear
	12 semi-pro	21 ± 2	183.4 ± 5.8	93.6 ± 8.5	10–20	4	3	Sprint + RT without feedback		
Shattock and Tee [45]	10 semi-pro	22 ± 3	-	93.1 ± 14.5	10–20–40	6	4	Velocity-based RT	Single-beam photocell timing system	Changes in sprint times for both the autoregulation methodologies were almost certainly trivial, and none of the changes could be considered significant
	10 semi-pro	23 ± 3	-	95.6 ± 16.8	10–20–40	6	4	Effort-based RT		
Lahti et al. [46]	10 pro	20 ± 1	190 ± 0.1	94.4 ± 9.1	5–20	8	2	Assisted sprint training	Radar device	Significant between-group difference only for 20 m sprint time, with actual sprint performance improvements only for resisted sprint group.
	6 pro	19 ± 0.3	183 ± 0.1	91.4 ± 15.3	5–20	8	2	Resisted sprint training		
Appleby et al. [47]	13 academy	21.8 ± 3.3	184.3 ± 5.9	101.3 ± 12.8	5–20	18	3	Bilateral + speed + skills + conditioning	Dual-beam electronic timing gates	Meaningful improvements for both groups over 5 and 20 m, without a clear difference between groups
	10 academy	23.1 ± 4.1	186.5 ± 5.1	104.6 ± 11.5	5–20	18	3	Unilateral + speed + skills + conditioning		
	10 academy	24.6 ± 5.3	183.2 ± 7.4	93.1 ± 10.4	5–20	18	3	Speed + skills + conditioning (control)		
Zabaloy et al. [48]	8 pro	21 ± 3	179 ± 9	84.4 ± 15.5	5–10–20–30	7	2	Non individualised training	Electronic timing gates	Training protocols based on FV profile imbalance provide greater benefits in sprinting than non-individualised training protocols, although all achieve significant improvements
	6 pro	21 ± 4	174 ± 7	84.1 ± 11.6	5–10–20–30	7	2	Velocity imbalance training		
	11 pro	24 ± 3	178 ± 5	89.4 ± 11.1	5–10–20–30	7	2	Force imbalance training		
	9 pro	22 ± 4	178 ± 7	93.5 ± 15.6	5–10–20–30	7	2	Well-balanced training		
Simpson et al. [49]	15 elite	24 ± 3	181.3 ± 6	94.9 ± 21.6	10–20	8	3	Optimised experimental group	Electronic timing gates	In none of the cases are adaptations in the maximum speed at 10 and 20 m achieved
	14 elite							Non-optimised experimental group		
Sinclair et al. [50]	13 pro	18.8 ± 0.6	182.5 ± 6.1	89.5 ± 11.4	5–10–20	8	2	Sprinted-based group	Electronic timing gates	For sprint-based outcomes, although both groups improved significantly, there were no statistical differences between the two training methods
	13 pro	18.9 ± 0.5	181.8 ± 5.1	85.7 ± 11.5	5–10–20	8	2	Sled group		
Scott et al. [51]	8 university	20.3 ± 1	178 ± 8.7	84.7 ± 10.6	5–10–15–20	6	2	Variable-resistance CPX	Electronic timing gates	Both variable-resistance training and traditional complex training provided similar improvements in sprinting, being better than the control group but with no differences between them
	8 university	22.8 ± 3.6	185 ± 4.7	96.2 ± 10.4	5–10–15–20	6	2	Traditional CPX		
	8 university	26 ± 4	181 ± 6.9	92.2 ± 10	5–10–15–20	6	2	Control		
Loturco et al. [52]	14 elite	25.4 ± 2.7	182 ± 0.15	94.5 ± 16.4	30	4	3	Light-load jump squat training	Photocell timing system	No significant differences were found between the two groups except that the low-load group experienced less fatigue after training. However, certain improvements in performance were noted in both cases without reaching significance
	11 elite							Heavy-load jump squat training		

Note: y = years; cm = centimetres; kg = kilograms; m = metres; pro = professional; RT = resistance training; GPP = general physical preparation; HIIT = high-intensity interval training; RHIE = repeated high-intensity effort; AEL = accentuated eccentric loading; CPX = complex training.

## Data Availability

The raw data supporting the conclusions of this article will be made available by the corresponding or last authors of the manuscript on request.
